# Do Mechanical and Physicochemical Properties of Orthodontic NiTi Wires Remain Stable In Vivo?

**DOI:** 10.1155/2016/5268629

**Published:** 2016-12-14

**Authors:** Michał Sarul, Małgorzata Rutkowska-Gorczyca, Jerzy Detyna, Anna Zięty, Maciej Kawala, Joanna Antoszewska-Smith

**Affiliations:** ^1^Department of Dentofacial Orthopedics and Orthodontics, Wroclaw Medical University, Krakowska Street 26, 50-425 Wroclaw, Poland; ^2^Department of Mechanics, Materials Science and Engineering, Wroclaw University of Technology, Smoluchowskiego Street 25, 50-370 Wroclaw, Poland; ^3^Department of Dental Prosthetics, Wroclaw Medical University, Krakowska Street 26, 50-425 Wroclaw, Poland

## Abstract

*Introduction and Aim.* Exceptional properties of the NiTi archwires may be jeopardized by the oral cavity; thus its long-term effect on the mechanical and physiochemical properties of NiTi archwires was the aim of work.* Material and Methods*. Study group comprised sixty 0.016 × 0.022 NiTi archwires from the same manufacturer evaluated (group A) after the first 12 weeks of orthodontic treatment. 30 mm long pieces cut off from each wire prior to insertion formed the control group B. Obeying the strict rules of randomization, all samples were subjected to microscopic evaluation and nanoindentation test.* Results.* Both groups displayed substantial presence of nonmetallic inclusions. Heterogeneity of the structure and its alteration after usage were found in groups B and A, respectively.* Conclusions.* Long-term, reliable prediction of biomechanics of NiTi wires in vivo is impossible, especially new archwires from the same vendor display different physiochemical properties. Moreover, manufacturers have to decrease contamination in the production process in order to minimize risk of mutual negative influence of nickel-titanium archwires and oral environment.

## 1. Introduction

Since the 18th century when Edward Angle introduced orthodontic fixed appliances based on physical properties of wires inserted into bracket slots, they have been constantly improved, thus facilitating both the orthodontist's work and efficiency of the devices [1].

Considering composition of wires 3 major alloys may be currently distinguished: stainless steel, *β*-titanium, and—last but not least—nickel-titanium ones. Exceptional flexibility of the latter ones, resulting from their physical and chemical properties, technically allows application of nickel-titanium archwires thorough the whole therapy, often limiting number of used wires to 2-3 per treatment [1–4]. Nonetheless oral environment, namely, repetitive, wide-range changes of temperature; low pH; increased partial pressure of hydrogen ions as well as the microbe metabolism products, altogether may have vital effects on the wires via changing their physical and chemical properties [3, 5–9]. Our previous studies showed that the oral environment can change the mechanical properties of nickel-titanium wires within a period as short as 6–8 weeks [5]; that is why the obvious questions have risen. (1) Is the long-term influence of an oral environment capable of changing microstructure of nickel-titanium wires? (2) Do these changes possibly affect the release of microelements into the oral environment? Resolving those yet not answered issues might have brought scientific evidence supporting either health-care—in terms of prevention against undesirable chemical influences—or production of high quality archwires maintaining their properties thorough the orthodontic treatment course.

Thus this metallographic study was aimed at assessing the long-term effect of oral environment on NiTi wires in order to answer those above posted questions.

## 2. Material and Methods

**Figure 1:**
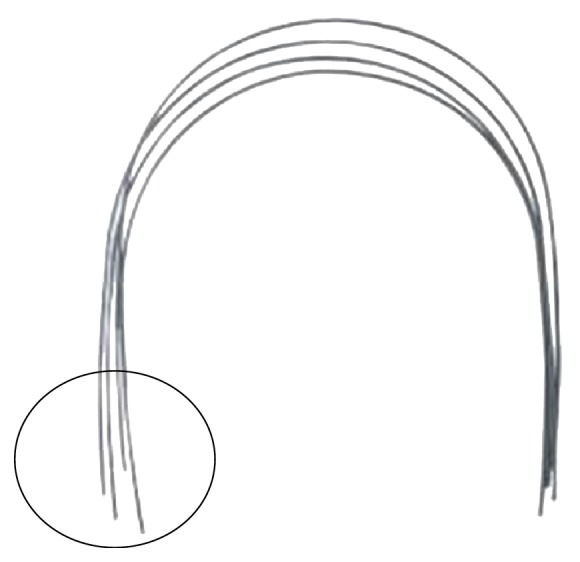
Orthodontic wires: location of the specimen at the distal free ends is marked with the circle.

In order to increase homogeneity 60 orthodontic patients undergoing treatment with the products from only one company were enrolled in the project. Simultaneously the choice of one vendor allows obtaining preliminary data that will allow authors to expand research in the direction of cross-sectional studies on the wires of other brands. Study group (A) comprised 0.016 × 0.022 NiTi archwires randomly taken from 12 different packages, again originating from the same facility, passively, and piggyback ligated to the levelling archwires. Prior to ligation 30 mm long distal ends (Figure 1) were cut off from each archwire, inserted into the plastic bags, numbered consecutively from B1 to B60, and stored in the patient's paper file thus, composing control group B. After the first 12 weeks of orthodontic treatment 30 mm long pieces of each wire from group A were cut off and inserted separately into the plastic bags numbered from A1 to A60 according to the samples from group B.

Two envelopes containing all wires from both groups marked “A” and “B” for blinding the outcomes were sent to the laboratory. Successively the engineer further treated all the specimens grinding them in the longitudinal and transverse manner depending on the direction of plastic deformation and then polishing them mechanically. All samples were subjected to microscopic evaluation and nanoindentation test. Afterwards randomly selected half of the wires from group A and B separately underwent chemical etching with a mixture of hydrofluoric and nitric V acids (1.5 : 1 ratio) and were subjected to microscopic analysis.

Composition of the whole material is displayed in Table 1.

## 3. Microscopic Analysis

Visitron Systems integrated digital camera using NIS Elements BR software registered all specimen images further evaluated under NIKON ECLIPSE MA200 microscope with magnification ranging from 100 to 1000 times.

In order to assess the degree of contamination with nonmetallic inclusions the microscopic picture of the nonetched samples was compared with the norm PN-64/H-04510. Its scale contains 5 patterns, each of them divided into three variants (a, b, and c) based on the distribution of nonmetallic inclusions. When providing the final results only the highest scored pattern counts were registered separately for each type of inclusion. Thus the contamination is pronounced as the number of pattern and its variety.

Microscopic analysis also enabled us to determine crystal structure of all etched samples in the study material.

## 4. Nanohardness Analysis

Indentation Release Candidate “SBO” allowed measurement of the nanohardness based on maximum operating force and the maximal probe indentation (HV_IT_) as well as on Instrumental Young's Modulus (*E*_IT_) calculations. The measurement itself was nothing else but the maximum 250.0 mN loading of Berkovich indenter, lasting 15 seconds and resulting in the tetrahedron shape imprint. Both force values and the depth of penetration of the blade were recorded in the cycle of loading and unloading; an arithmetic mean was considered to be the end results. Loading factors as a function of penetration depth were determined for each cycle in three randomly selected locations of the wires from groups A and B separately.

Instrumental Young's Modulus was calculated using the Oliver and Pharr's method determining the force-displacement curve applying an appropriate formula:

(1) $$\begin{array}{c} \frac{1}{E_{IT}*}=\frac{\left(1-v^{2}\right)}{E_{IT}}+\frac{\left(1-{vi}^{2}\right)}{E_{i}}. \end{array}$$

*v* is sample Poison's fraction, *vi*: Poison's fraction taking into account the indenter, and *E*_IT_: Instrumental Young's Modulus.

## 5. Statistical Analysis

Normality and homogeneity of variance were preanalysed with Levene's test, which verified positive assumptions for implementation of parametric testing. The* t*-test applied was to evaluate intra- and intergroup differences of nanohardness in both groups.

Statistical significance level was established at *p* < 0.05.

## 6. Results

### 6.1. Microscopic Analysis of Nonetched Samples

Microscopic images of the specimens showed substantial presence of nonmetallic inclusions, mainly in the form of silicates and oxides. The silicate inclusions were arranged in chains amounted from 1 to 3 and mainly displayed 3a pattern in group A and 1a and 2b patterns in group B. Oxidant impurities were arranged in dots amounted from 2 to 5 and mainly displayed pattern 5a in group A and 2a and 5a patterns in group B.

### 6.2. Microscopic Analysis of Etched Samples

**Figure 2:**
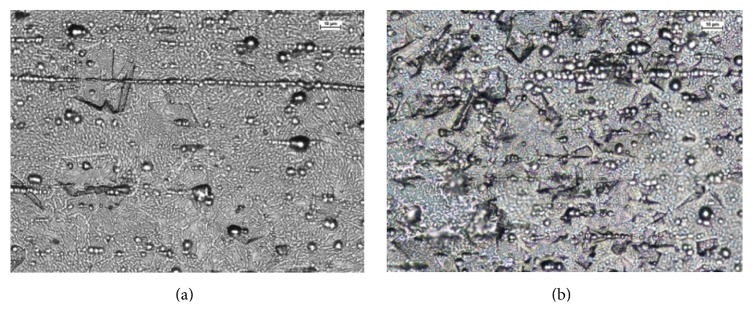
Microstructure of etched material (a) used wires with predominant martensitic phase and (b) new wires with fine-grained austenitic structure.

The wires from group A presented combined structures, however, dominated by martensitic areas in all samples (Figure 2(a)). This arrangement of the microstructure indicates that the 12-week lasting influence of oral environment facilitates the transition of internal crystal structure into harder phase. The wires from group B showed austenite structure with grains diameter ranging from 0.5 to 1.5 *µ*m (Figure 2(b)). Small martensitic areas are the evidence of partial transformation present already in the brand new products.

### 6.3. Nanohardness Analysis

**Figure 3:**
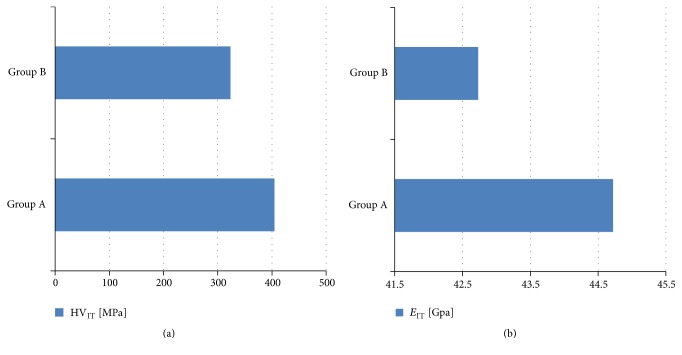
The values obtained in group A (used wires) and group B (new wires): (a) nanohardness (HV_IT_) and (b) *E*_IT_.

The results were in accordance with microscopic evaluation. Statistically significant (*p* < 0.05) increase of nanohardness in the used archwires (group A) was apparent: mean HV_IT_ and *E*_IT_ values exceeded the ones in group B by 100 MPa (Figure 3(a)) and nearly 3 GPa (Figure 3(b)), respectively. It is worth to emphasize that HV_IT_ and *E*_IT_ parameters in groups A and B and in different points of the measurements displayed significantly diverse values (*p* < 0.05). This may indicate both the heterogeneity of the single wire and the presence of individual components in the structure of the NiTi alloy.

**Figure 4:**
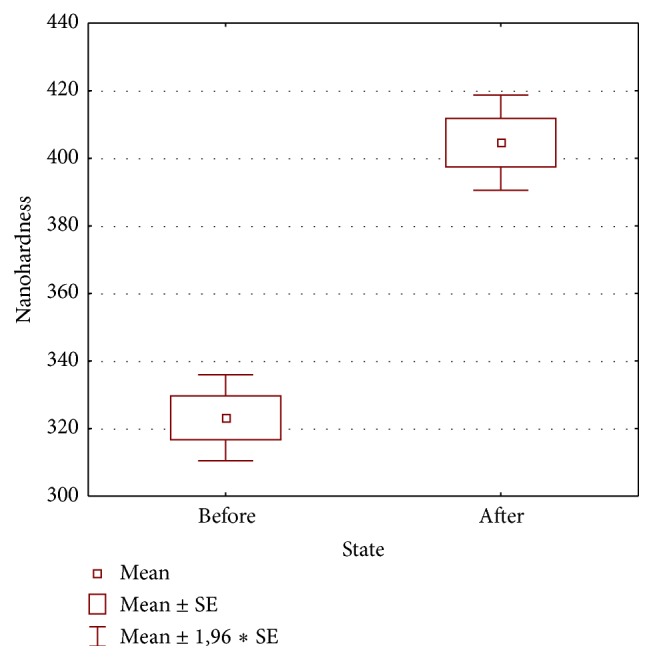
Box plot of nanohardness mean values in group A (used) and group B (new) wires; SE: standard error.

Results of statistical analysis are shown in Table 2 and in Figure 4.

## 7. Discussion

Orthodontic biomechanics is based—among others—on the assumption that aligning and levelling archwires produce long-term, light, and constant force values [10]. Nonetheless our previous studies have already proved that those forces are likely to change with the function of time and are not always predictable [11]. Presented research was aimed at identifying alterations in the structure of orthodontic wires after their 12-week acting in vivo. It brought the evidence that oral environment evidently affects NiTi wires changing their structure and thereby their properties, which is in accordance with the results obtained by other clinicians [8, 9, 11].

Surprisingly the wires examined in the current research exhibited both uneven structure and mechanical properties already at the stage of their postproduction. Microscopic evaluation and nanoindentation tests showed that all new wires from control group presented with austenite and martensitic phases although the previous ones prevailed in all specimens. Such results allow assumption that since the internal configuration of new 0.016 × 0.022 NiTi wires of the same manufacturer varies from sample to sample and within the samples themselves, comparison with the wires produced in another facility may bring even more profoundly diverse results. It seems to be fully justified when analysing the results obtained by Nakano et al., Parvizi and Rock, and Nikolai who reported very high standard deviation in the force-deflection plot of brand new nickel-titanium wires from different vendors [12–14]. Due to the lack of studies evaluating nanohardness of nickel-titanium orthodontic wires one can only presume that their inconsistent crystal structure and diverse mechanical properties may subsequently affect biomechanics.

Such phenomena immediately arises further question: do NiTi wires of the same manufacturer change their properties and/or structure and do they change it in the same manner during an orthodontic treatment? Our results obtained in previous [11] and current studies allow answering both posted queries: the results of nanohardness significantly increased under influence of the oral environment (*p* < 0.05), although in the uniform manner (*p* > 0.05 for intragroup A comparison). Presence of permanently and diversely altered crystal structures within the same used wire is substantial. Such lack of uniformity in transformation of the internal net of atoms is an evident and major drawback impairing mechanical properties of the NiTi wires. It may be summarized that heterogeneous crystal structure negatively influencing mechanical properties, which exists already at the postproduction stage, is subjected to further negative changes under the influence of the oral environment, although significance and clinical impact of this finding require further investigation.

The presented results demonstrated no substantial differences between new and used wires in regards to the amount of nonmetallic inclusions, although impurities such as silicate and oxidant inclusions were present more frequently in group A. However, it is known already that their vestigial presence provokes deterioration of mechanical properties of wires and hence makes prediction of their exact mechanical and electrochemical behaviour technically difficult or even impossible [14–18]. Furthermore, since the orthodontic wires are used in the environment of the human body their composition should be extremely uniform and consistent with the data reported by the manufacturer [18, 19]. Whether the content of such inclusions results in an increased susceptibility to corrosion and/or cracking [20, 21] remains still an open question. However nonmetallic impurities present already at the postproductive stage and increased under influence of the oral environment must not be neglected.

## 8. Conclusions

- The results proved that the NiTi wires produced by the same manufacturer do not have equal physical properties already at the stage of postproduction and that their structure is further altered by the long-term influence of an oral environment. Therefore reliable prediction of their biomechanics is impossible.
- Since both used and new archwires contain a substantial amount of nonmetallic inclusions none of them meets the requirements for medical materials, since all impurities may be released to the oral cavity. The nonmetallic inclusions may also provoke corrosion or cracking of the wires, thus possibly affecting an orthodontic treatment; nevertheless solving this issue requires additional studies.
- Manufacturers have to improve their production process in order to minimize mutual negative influence of nickel-titanium archwires and oral environment.

## Figures and Tables

**Table 1:** Composition of the study material.

Group	Nonetched specimen (*n*)	Type of analysis	Etched specimen (*n*)	Type of analysis
A (used)	ANE (60)	Microscopic	AE (30)	Microscopic
		Nanoindentation		
B (brand, new)	BNE (60)	Microscopic	BE (30)	Microscopic
		Nanoindentation		

**Table 2:** Results of *t*-test comparing the average value of nanohardness in groups A and B.

	Group A	Group B
HV_IT_ (MPa: mean value ± SD)	404.62 ± 40.3	323.18 ± 8.7
*p* (intragroup analysis)	0.03258	0.006258
*t* value	−7.01238	
*p* (intergroup analysis)	0.00	
*E*_IT_ (GPa: mean value ± SD)	44,72 ± 1,0	42,73 ± 1.8
*p* (intragroup analysis)	0.01246	0.00246
*t* value	3,70801	
*p* (intergroup analysis)	0.000996	

*p*: the level of significance, *t* value: the difference coefficient, and SD: standard deviation.
